# (Near) Real-Time Snow Water Equivalent Observation Using GNSS Refractometry and RTKLIB

**DOI:** 10.3390/s22186918

**Published:** 2022-09-13

**Authors:** Ladina Steiner, Géraldine Studemann, David Eugen Grimm, Christoph Marty, Silvan Leinss

**Affiliations:** 1 Alfred-Wegener-Institut Helmholtz-Zentrum für Polar- und Meeresforschung (AWI), 27515 Bremerhaven, Germany; 2 Institute of Geomatics, University of Sciences and Arts of Northwestern Switzerland (FHNW), 4132 Muttenz, Switzerland; 3 WSL Institute for Snow and Avalanche Research SLF, 7260 Davos, Switzerland; 4 ETH Zurich, 8093 Zurich, Switzerland

**Keywords:** global navigation satellite systems, GNSS, refractometry, snow water equivalent, SWE, real time, RTK, RTKLIB, emlid

## Abstract

Global navigation satellite system (GNSS) refractometry enables automated and continuous in situ snow water equivalent (SWE) observations. Such accurate and reliable in situ data are needed for calibration and validation of remote sensing data and could enhance snow hydrological monitoring and modeling. In contrast to previous studies which relied on post-processing with the highly sophisticated Bernese GNSS processing software, the feasibility of in situ SWE determination in post-processing and (near) real time using the open-source GNSS processing software RTKLIB and GNSS refractometry based on the biased coordinate Up component is investigated here. Available GNSS observations from a fixed, high-end GNSS refractometry snow monitoring setup in the Swiss Alps are reprocessed for the season 2016/17 to investigate the applicability of RTKLIB in post-processing. A fixed, low-cost setup provides continuous SWE estimates in near real time at a low cost for the complete 2021/22 season. Additionally, a mobile, (near) real-time and low-cost setup was designed and evaluated in March 2020. The fixed and mobile multi-frequency GNSS setups demonstrate the feasibility of (near) real-time SWE estimation using GNSS refractometry. Compared to state-of-the-art manual SWE observations, a mean relative bias below 5% is achieved for (near) real-time and post-processed SWE estimation using RTKLIB.

## 1. Introduction

The estimation of the spatial distribution of the water equivalent of the snow cover (SWE) in mountain catchment areas is presently the most important unsolved challenge in snow hydrology [1]. In situ data is important for calibration and validation of extensive remote sensing observations and snow hydrological modeling [2,3,4]. The works of [5,6] give a detailed description of various ground-based techniques for observing the SWE.

**Figure 1:**
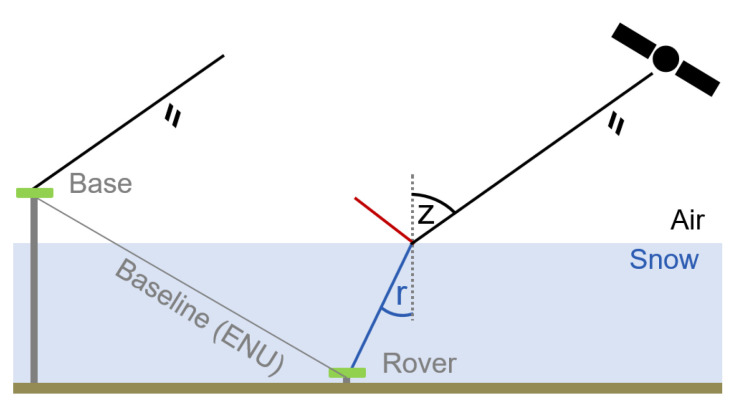
Simplified schema illustrating a fixed GNSS refractometry setup. The base antenna, mounted on a pole above the snowpack serves as reference. The rover antenna is installed on the solid ground and covered by snow. The GNSS signals are reflected (red) and refracted (blue) at the air/snow boundary and depend on the incidence (z) and thus refractive (r) angles. The SWE is derived from the biased GNSS baseline Up component due to the influence of the snow above the rover antenna.

Global navigation satellite system (GNSS) refractometry permits accurate and continuous in situ SWE monitoring [7,8]. A GNSS reference (base) antenna is mounted above the snowpack, while a second GNSS (rover) antenna is placed on the ground where it is continuously covered by snow accumulation (Figure 1). Observed GNSS signals are refracted at the air/snow boundary (and between layers within the snowpack) and delayed while propagating through the snowpack, depending on the SWE above the buried GNSS antenna.

In contrast to manual [9,10] point-wise in situ SWE observations, GNSS-refractometry-based SWE monitoring is non-destructive, needs little effort and cost for installation and maintenance, and provides high temporal resolution. Compared to automatic snow depth sensors, the GNSS refractometry method directly estimates the SWE without the need for very scarce and laborious density observations for the conversion of snow depth to SWE [11]. The spatial footprint of GNSS refractometry depends on the permittivity and the snow depth of the snowpack overlying the buried GNSS antenna and the angle of incidence of the GNSS signals [12]. For the most accurate GNSS processing, available GNSS signals are tracked from all elevations and azimuths. In case of dry snow, the spatial footprint of GNSS refractometry varies from 0.045 $m^{2}$ to 41 $m^{2}$ (0.2 m to 7.2 m in diameter) for snow depths above the buried GNSS antenna of 0.1 m and 3 m. For wet snow, the spatial footprint is much smaller with 0.012 $m^{2}$ to 11 $m^{2}$ (0.1 m to 3.7 m in diameter) for snow depths of 0.1 and 3 m. The representativeness of GNSS refractometry using a single setup is thus limited to a point-wise scale.

The temporal and spatial resolution is comparable with automatic SWE observations by buried snow pillows, scales, ground radar, cosmic ray, and acoustic snow gauges [13,14,15,16,17,18,19,20]. The main advantage of GNSS refractometry compared to these buried automatic SWE observation sensors is the very low cost and size of the system, as well as the global availability and coverage of GNSS signals, enabling the permanent installation of several distributed buried GNSS sensors at a low cost. Additionally, a mobile GNSS refractometry setup enables distributed SWE measurements at relatively high speeds and low cost, increasing the spatial footprint of this method. Due to the high spatial and temporal variability of the SWE at small to regional scales, several distributed in situ SWE observations from intermediate scales of several hundred meters (e.g., mountain catchment areas) are of high value to complement regional-scale snow modelling and remote sensing products [21].

Following the idea of [7,22], a SWE estimation model was developed by [12] based on the Global Positioning System (GPS) phase signal path delay due to a present single water layer of depth *d*, being equivalent to the SWE above a submerged/buried GNSS antenna. Refs. [23,24] demonstrated the successful daily and hourly SWE estimation over three complete (dry and wet snow) seasons using refracted GPS signals. Single-frequency GPS (GPS L1) data collected by high-end GNSS sensors were thereby thoroughly analyzed using the scientific Bernese GNSS processing software. Refs. [25,26] used a similar, phase-based, approach for SWE estimation with low-cost GPS L1 sensors. The use of low-cost sensors for hourly SWE retrieval based on GNSS refractometry was independently verified by [27] for a complete dry and wet snow season. In these studies, the SWE was introduced and estimated as an additional parameter in the GNSS phase observation equation. The impact on the coordinate solution in case of a buried GNSS antenna was demonstrated for the first time by [24]. A strong correlation (99.7%) between the SWE and the coordinate Up component was revealed, illustrating the feasibility of using the Up component, biased due to the overlying snowpack, for SWE estimation.

In contrast to previous studies, the present study investigates the feasibility of high-temporal (near) real-time in situ SWE determination by GNSS refractometry. Multi-frequency and multi-system GNSS observations from newly available (since 2020) multi-band low-cost GNSS sensors are used to retrieve the SWE based on the estimation of the bias in the coordinate Up component. With this method, no additional SWE parameter is needed in the GNSS observation equation, and the SWE can be directly estimated using standard, open-source GNSS processing software, which significantly simplifies the implementation. For the first time, GNSS refractometry can thus be applied in (near) real time directly in the field using real-time kinematic (RTK) GNSS refractometry in a fixed and mobile low-cost snow monitoring setup.

An overview about fixed and mobile GNSS refractometry snow monitoring setups and available reference data is given in Section 2. Section 3.1 summarizes the methodology. The data processing is specified in Section 3.2. Section 4 delineates results of post-processing in Section 4.1 and (near) real-time SWE estimations from a fixed setup in Section 4.2 and a mobile setup in Section 4.3. Section 5 provides a discussion and conclusion.

## 2. GNSS Refractometry Snow Monitoring Setups

GNSS refractometry snow monitoring systems were set up at two different study sites for investigating the feasibility of GNSS refractometry for SWE estimation using the standard open-source RTKLIB software in post-processing (15 min resolution) and (near) real time. For the fixed, low-cost setup and near real-time processing, the GNSS baseline was estimated with a temporal resolution of 1 Hz and subsequently filtered by a moving median over 24 h and resampled to 10 min. For the (near) real-time processing from the mobile, low-cost setup, the GNSS baseline was determined directly in the field over a 15 min observation window.

The fixed setups were installed on stable ground during the snow-free period to monitor changes in the SWE over time at a specific location. Data were stored or processed in real time. Due to the fixed location, the estimated SWE time series can be filtered subsequently which leads to enhanced SWE results. The fixed setups have a high temporal, but a low spatial resolution due to the point-wise measurements. To increase the spatial resolution, the mobile setup was developed. This lightweight and low-cost mobile setup was designed to enable SWE measurements in snow-covered areas at different locations in near real time. Little effort is needed as the small-sized system fits in a hole drilled into the snowpack, making the measurement additionally fast compared to traditional snow tube samplers where digging snow pits is a labor-intensive prerequisite, especially in deep snowpacks. Due to the availability of the SWE result directly in the field, SWE measurements are possible in different snow depths in the hole to indirectly derive layered density information and/or in different spatial locations to estimate the spatial heterogeneity of the SWE.

### 2.1. Fixed, High-End Setup for Post-Processing

GNSS refractometry data from the Davos Weissfluhjoch test site at 2536 m a.s.l., operated by the WSL Institute for Snow and Avalanche Research SLF (WSL SLF), are available for the season 2016/17 from a previous study [23]. The high-end GNSS reference station (Leica GR10 receiver, Leica AS10 antenna) was deployed on a pole at a 5.3 m height. A high-end GNSS antenna (Leica AS10) was mounted on the ground below and connected to a high-end GNSS receiver (Leica GR10). Raw GNSS observations (phase, code, signal-to-noise) are available with a 1 Hz sampling rate for GNSS post-processing. Pressure based snow scale measurements (30 min) and biweekly manual snow tube observations from the test site (10–20 m next to the GNSS sensors) serve as ground truth.

Multi-frequency and multi-system GNSS data collected from this fixed, high-end GNSS refractometry snow monitoring setup is publicly available at [28] and reprocessed to evaluate the feasibility of post-processed SWE estimation using RTKLIB (Section 3.2.1 and Section 4.1).

### 2.2. Fixed, Low-Cost Setup for Near Real-Time Processing

**Figure 2:**
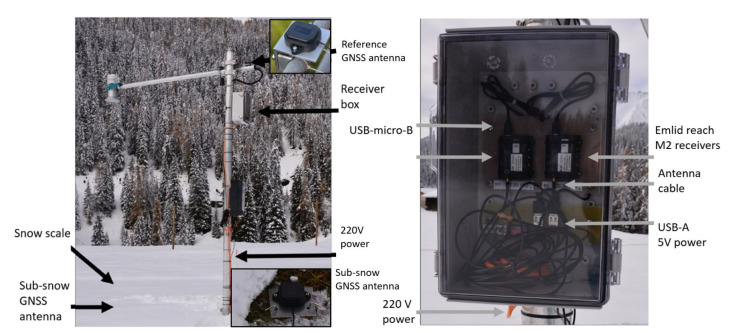
Davos Laret site equipped with the fixed, low-cost GNSS refractometry snow monitoring system and a snow scale. Low-cost multi-frequency and multi-system U-blox ANN-MB-00 antennas and Emlid Reach M2 receivers were deployed for near real-time processing.

For the first time, a near real-time (1 Hz) GNSS refractometry snow monitoring system was set up and installed at the WSL SLF Davos Laret test site at 1500 m a.s.l. (Figure 2). The test site is located in a North–East to South–West facing mountain valley and surrounded by high mountains. Ground-truth data is provided by a close-by snow scale with 10 min sampling rate and weekly manual snow tube observations, both within a distance of 5 m to the GNSS refractometry setup.

The fixed, low-cost GNSS refractometry snow monitoring setup is composed of two Emlid Reach M2 receivers and two u-blox ANN-MB-00 antennas (Figure 2). These user-friendly instruments allow multi-frequency and multi-system GNSS observations at a low cost, low weight, minimal size, and high precision. The GNSS reference antenna is mounted on a pole at 2.8 m height with a very short GNSS baseline to the sub-snow antenna which is fixed on the ground. The system was installed in November 2021 after the first snow fall and operates in RTK GNSS mode. Estimated real-time (1 Hz) GNSS baselines, in East, North, and Up components, were recorded from November 2021–April 2022 and are publicly available at [29]. Due to a system outage at the base receiver on 13 April, no baselines are available for the last week of the season during the snowmelt. The near real-time feasibility of GNSS-refractometry-based SWE monitoring using the fixed, low-cost setup is assessed for the most recent 2021/22 season at Davos Laret (Section 3.2.2 and Section 4.2).

### 2.3. Mobile, Low-Cost Setup for (Near) Real-Time Processing

**Figure 3:**
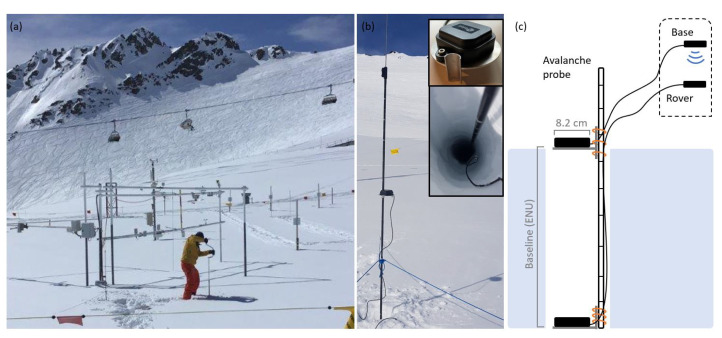
Mobile, low-cost GNSS refractometry snow monitoring system setup at Davos Weissfluhjoch: (**a**) drilling a 11.2 cm hole in the snowpack; (**b**) system deployed in the field. The base antenna is located above the snow surface, whereas the sub snow antenna is placed on the ground below the snowpack. A string with small anchors fix the system while measuring; (**c**) schematic setup illustrating the GNSS base and rover antennas (u-blox ANN-MB-00) attached to an avalanche probe by cable ties. The true baseline between both antennas can directly be read out by the metric scale on the avalanche probe. The receivers (Emlid Reach M2) are connected to the antennas by cable and can be stored, e.g., in a backpack. The system is configured in RTK GNSS mode where the base data is sent to the rover via the internal Wi-Fi.

A mobile GNSS refractometry snow monitoring setup was designed and consists of two low-cost multi-frequency and multi-system GNSS antennas (u-blox ANN-MB-00) mounted on an avalanche probe (Figure 3b,c) and connected to the low-cost multi-frequency and multi-system GNSS receivers (Emlid Reach M2). The antennas were mounted on a 5 mm thick aluminum plate with 8.3 cm in diameter. The plate was attached to the probe by a welded half tube of 3.5 cm length and cable ties, allowing us to move the upper base antenna and place it above the snowpack. The lower rover antenna was mounted at the bottom of the probe so that the antenna could be placed on the ground to estimate the bulk SWE, averaged over an area of several square meters around the antenna. The lightweight setup is portable and delivers in situ SWE estimates directly in the field. To place the rover antenna below the snow, a vertical hole was drilled with an 11.2 cm diameter ice drill (Kogha Arctic). Drilling a hole (Figure 3a) proved to be significantly faster and less destructive compared to digging a snow pit. Furthermore, snow pits can affect the signal propagation in the cone above the rover antenna which, in turn, deteriorates the SWE estimates.

The system is configured in RTK GNSS mode (Section 3.2.3) where the base receiver transmits the base position and all GNSS observations to the rover via an internal Wi-Fi. To initialize the system, both antennas need to be above the snowpack. After the rover initialized, the RTK solution state needs to be fixed, indicating that the float phase ambiguities are resolved to integer values. The rover was then slowly lowered to the bottom of the drilled hole. It is important to keep the fixed RTK solution which could be lost by lowering the rover antenna too fast in the snowpack. In this case, the system needs to be elevated above the snowpack until the ambiguities are fixed. Reducing the signal-to-noise (SNR) mask in the receiver configuration improves the RTK solution stability within the snowpack as GNSS signal strengths are decreased while propagating through snow [12]. The Emlid Reach M2 can be connected via the built-in Bluetooth or Wi-Fi which allows us to control and configure the receivers via a smartphone or a tablet. The receivers are accessed through the Emlid ReachView mobile application, allowing us to read out the baseline and the coordinates of both antennas directly in the field and to configure or monitor the receiver settings and state.

The new mobile, low-cost GNSS refractometry snow monitoring setup was tested for its (near) real-time SWE estimation feasibility (Section 4.3) at Davos Weissfluhjoch on 26 March 2021 in approximately 2.5 m deep snow close to the fixed, high-end GNSS refractometry setup (Section 2.1). Two holes were drilled 3 m next to each other and measured three times each for 15 min. A snow profile of 31 March 2021 from the same test site (10–20 m next to the mobile experiment) served as reference. The snowpack settled between both dates without new snow accumulation, influencing only the snow density, not the bulk SWE.

## 3. Method

In the present study, the SWE of the snowpack overlying the buried GNSS rover antenna was estimated using the GNSS refractometry method based on the bias in the coordinate Up (height) component [24].

### 3.1. GNSS Refractometry Based on the Biased Up Component

Introducing the GNSS signal path delay $\delta L_{w}$ as an additional parameter in the GNSS phase observation equation (Equation (1); [30]) enables us to precisely estimate the SWE by knowing and fixing the coordinates of the base and rover station [23]:

(1) $$L_{A}^{j}=\rho_{A}^{j}+\delta \rho_{A}^{j}+\delta L_{w}+\lambda N_{A}^{j}+\sigma_{A}^{j}$$

Thereby, $L_{A}^{j}$ is the observed carrier phase measurement in meter, and $\rho_{A}^{j}$ is the geometric distance between the GNSS antenna *A* and a satellite *j*, impacted by the atmospheric, relativistic, and multipath delays, as well as the satellite and receiver clock biases $\delta \rho_{A}^{j}$; $\lambda N_{A}^{j}$ represents the ambiguity phase bias and $\sigma_{A}^{j}$ the measurement noise. The differential GNSS processing to the close-by reference station mitigates the atmospheric (tropospheric and ionospheric) and relativistic effects and the satellite clock errors. The impacts of the antenna phase center offsets and variations are minimized by using similar GNSS antenna types with identical orientations for the base and the rover.

Instead of introducing and estimating the additional SWE parameter ($\delta L_{w}$) in the GNSS observation equation, the height bias of the GNSS rover antenna coordinate due to the snow cover was estimated in the present study. The SWE above the buried GNSS antenna was then derived from the bias in the estimated GNSS baseline Up component. The difference in the station height *h* or the Up (height) component of the baseline were strongly correlated with the SWE [24].

Assuming a single water layer model [12], the path delay $\delta L_{w}$, introduced by the signal propagation in water is collinear to the mapping function of the GNSS station height (*h*) and the receiver clock ($\delta t_{A}$) parameters [8,24]:

(2) $$\delta L_{w}=\mathrm{SWE}\cdot \left(\sqrt{n_{w}^{2}-\sin^{2}z}-\cos z\right)=\mathrm{SWE}\cdot F\left(z\right)$$

The path delay $\delta L_{w}$ depends on the SWE above the buried GNSS antenna, the refractive index of the representing single water layer $n_{w}^{2}$, and the observation zenith angle *z*. F(z) represents the mapping function to scale the SWE observation bias depending on the observed zenith angles.

The $\sqrt{n_{w}^{2}-\sin^{2}z}$ part of F(z) is almost constant due to $n_{w}=9.24$ and varies only up to 0.06 between $z=0^{\circ }$ and $z=90^{\circ }$. This part can thus be absorbed by the constant station clock parameter ($c\delta t_{A}$).

(3a) $$\frac{dL}{d(c\delta t_{A})}=1$$

(3b) $$\frac{dL}{\mathrm{dh}}=-\cos z$$

The cosz dependent part of F(z) is absorbed by the station height coordinate by 99.9% [24]. The influence of the SWE can therefore be represented as a linear combination of the station clock and the height coordinate:

(4a) $$\delta \mathrm{SWE}\sqrt{n_{w}^{2}-\sin^{2}z}-\delta \mathrm{SWE}\cos z\approx c\delta t_{A}-\delta h\cos z$$

(4b) δSWE≈δh

The station clock error and thus the constant part of F(z) cancels out by double-difference GNSS processing. The change in the SWE above the sub-snow antenna can therefore be derived based on the change in the estimated height component (Equation (4b)) of the rover coordinate. Equivalently, the Up component of the estimated baseline between both antennas can directly be used.

### 3.2. Data Processing with RTKLIB

The GNSS refractometry method based on the bias in the coordinate Up component [24] was applied for post and (near) real-time SWE estimation using RTKLIB.

#### 3.2.1. Post-Processing

To test the performance of the open-source standard GNSS processing software RTKLIB, the available seasonal dataset of the fixed, high-end GNSS refractometry snow monitoring setup from Davos Weissfluhjoch (Section 2.1) was post-processed in RTKLIB (version 2.4.3 b34). The multi-band GNSS observations were processed in time intervals of 15, 20, 30, 40, 45, and 60 min. As no significant deviations in the estimated SWE time series were present for the different time intervals and least noise was observed for the shortest interval, results are only presented here for 15 min processing intervals. Observations are used from all elevations as the use of an elevation mask reduces the SWE estimation accuracy [24]. Appendix B.1 lists the command used for automated RTKLIB post-processing with the configuration file and python script uploaded to [31]. Outliers were removed in resulting SWE time series based on a statistical threshold (3σ) and filtered by a moving median over 24 h. The GNSS-derived SWE results were resampled to match the temporal resolution of the manual (daily) and snow scale (30 min) reference observations.

#### 3.2.2. Near Real-Time Processing

To evaluate the feasibility of near real-time SWE monitoring using GNSS refractometry, the fixed, low-cost GNSS snow monitoring system at Davos Laret operated autonomously in RTK GNSS positioning mode. RTKLIB was also used in real-time applications, as it was implemented in the Emlid Reach (M2) RTK receivers whereby the mobile ReachView application serves, among others, as a web interface for RTKLIB [32]. The GNSS baseline in East, North, Up (ENU) between the base and rover antennas was measured and logged continuously at 1 Hz sampling rate. The change in the estimated baseline Up component (stored in .ENU log files) was equivalent to the SWE. Similarly to Section 3.2.1, outliers were removed in the resulting SWE time series based on a statistical threshold (3σ) and filtered by a moving median over 24 h. As the system was installed after the first snow fall and the true physical baseline could not be measured (Section 2.2), the δSWE was corrected based on the first manual observation of the SWE. The GNSS-derived SWE results were resampled to match the temporal resolution of the manual (daily) and snow scale (10 min) reference observations.

#### 3.2.3. (Near) Real-Time Processing in the Field

The mobile, low-cost GNSS refractometry snow monitoring setup was also configured in RTK GNSS positioning mode to investigate the feasibility for (near) real-time SWE estimation directly in the field. The base station coordinates were determined and fixed over a 10 min observation interval in the field. For the absolute positioning of the measurement location, a virtual reference station (VRS) of the Swiss Positioning Service (swipos) was used to achieve an accuracy in the centimeter range.

Measurements of the mobile setup were triggered manually and the baseline in East, North, Up components was estimated over a 15 min observation interval. The estimated total baseline was directly displayed in the Emlid ReachView mobile application when connected to the rover receiver. Since the sub-snow GNSS antenna was mounted straight below the base antenna, the total baseline corresponded to the Up (height) component when the probe was held vertically.

The difference between the baseline, estimated with RTK GNSS refractometry to the actual baseline, measured physically on the avalanche probe, corresponds to the bulk SWE above the sub-snow GNSS antenna (Equation (5)).

(5) $$\mathrm{SWE}=h_{\mathrm{Probe}}-h_{\mathrm{GNSS}}$$

with $h_{\mathrm{Probe}}$ being the physically measured vertical distance between the base and rover antenna reference points and $h_{\mathrm{GNSS}}$ the estimated GNSS baseline Up component.

## 4. Results

SWE estimation results are presented for the post-processed (Section 4.1) fixed, high-end and (near) real time, fixed (Section 4.2) and mobile (Section 4.3) low-cost GNSS snow monitoring setups.

### 4.1. Post-Processed SWE Estimation

**Figure 4:**
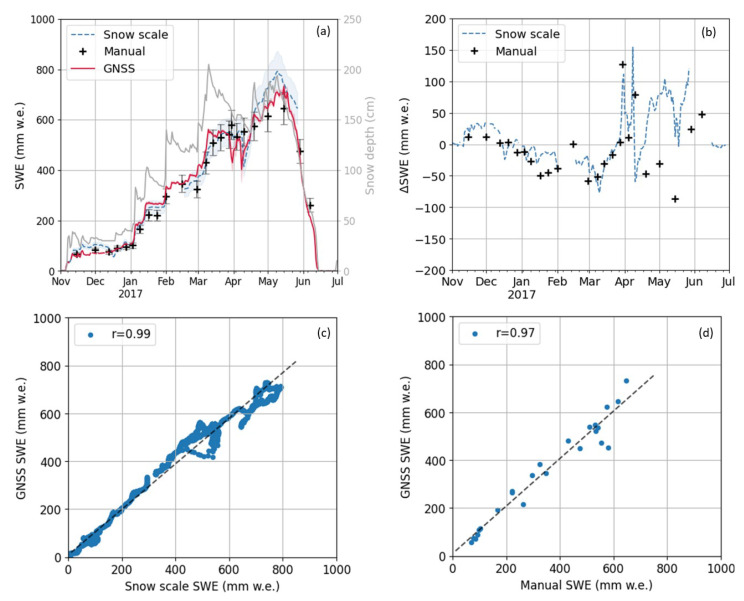
(**a**) Post-processed SWE overlaid with its standard deviation per day from the fixed, high-end GNSS snow monitoring setup at Davos Weissfluhjoch for 2016/17. A relative bias of 10% is added to the snow scale and manual observations. Daily snow depth is given in gray: (**b**) differences to reference sensors observations; correlation with (**c**) the snow scale; and (**d**) manual observations.

The post-processed SWE derived from the fixed, high-end GNSS snow monitoring setup at Davos Weissfluhjoch using the open-source software RTKLIB is shown in Figure 4a for November 2016 to July 2017. The median filtered SWE based on the biased GNSS baseline Up component (red) is overlaid by its standard deviation per day (transparent red), illustrating the SWE estimation noise (6.2 mm w.e) of the 30 s observations before filtering. The standard deviation of the estimated median filtered SWE time series is 0.2 mm w.e.

The estimated SWE is directly compared to reference data from close-by snow scale (blue) and manual observations (black) where a maximum SWE of approximately 800 mm w.e. is observed. The GNSS-derived SWE follows closely the trend observed by the reference sensors, with a root mean square error (RMSE) of 34.0 mm w.e. to the snow scale and 45.8 mm w.e. to the manual data. The mean relative bias (MRB) is below 10% compared to manual data and around 10% compared to the snow scale observations. Snow depth observations are illustrated for comparison and show strong settling of the snow after the accumulation of new snow, whereby the SWE is constant due to the increase in density. The GNSS-refractometry-derived SWE seems to detect snowmelt events, where the snow depth and the reference SWE decrease.

Differences to the snow scale and manual observations were below 70 mm w.e. for the dry snow period until April 2016 (Figure 4b). Afterwards, the SWE estimation differences increased up to 150 mm w.e. compared to the snow scale and manual observations. These reference sensors differ to each other in a similar order of magnitude as the GNSS-derived bias in the SWE estimation during the wet snow period. The reason is the uneven snow depth distribution within the test site as both sensors are approximately 20 m apart. A detailed description, including webcam pictures is given by [24]. Snow scale data was not available in June 2017 due to an instrument problem.

The post-processed SWE estimates are compared to the reference observations in Figure 4b,c using a linear regression. The GNSS-derived SWE estimates are highly correlated to the snow scale and manual measurements, with the Pearson cross-correlation coefficients (r) of 0.99 and 0.97, respectively. Small offsets (b) are present in the linear fit, and regression coefficients are summarized in Table 1.

The resulting SWE time series demonstrates the applicability of RTKLIB for SWE estimation in post-processing. It is thereby important that the ambiguities are resolved (fixed). Since RTKLIB does not offer the option to choose the GNSS solution type (e.g., standalone, float, or fixed), only fixed GNSS solutions need to be selected for further SWE estimation. Table 2 illustrates the pros and cons of RTKLIB regarding SWE estimation with GNSS refractometry.

### 4.2. Near Real-Time SWE Estimation

**Figure 5:**
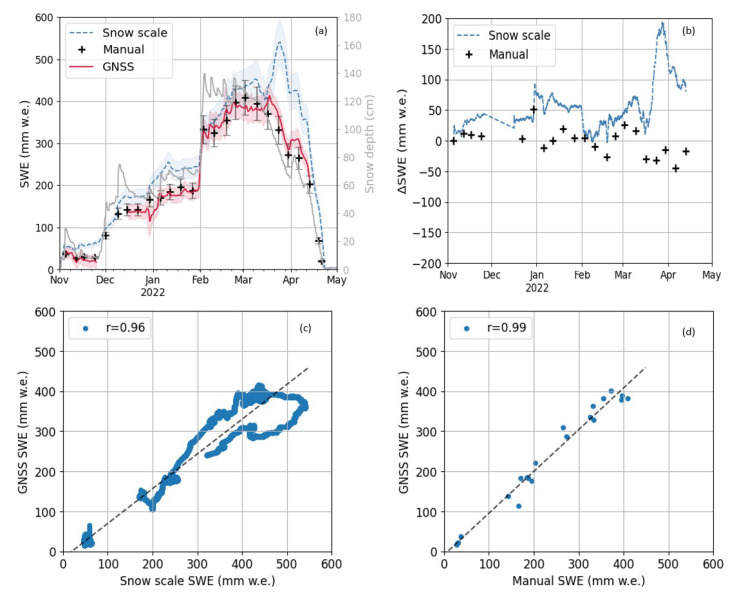
(**a**) Near real-time processed SWE overlaid with its standard deviation per day from the fixed, low-cost GNSS snow monitoring setup at Davos Laret for 2021/22. A relative bias of 10% is added to the snow scale and manual observations. Daily snow depth is given in grey: (**b**) differences to reference sensors observations; correlation with (**c**) the snow scale; and (**d**) manual observations.

The SWE time series derived by near real-time GNSS refractometry processing from the fixed, low-cost setup are illustrated in Figure 5a for 2021/22. The median filtered SWE based on the biased GNSS baseline Up component is overlaid by the standard deviation of all observations per day, representing the SWE estimation noise of the 1 Hz observations before filtering. These unfiltered SWE observations scatter with a mean standard deviation of 41 mm w.e. whereas the standard deviation of the estimated median-filtered SWE time series is 1.3 mm w.e.

Only fixed RTK solutions are selected and illustrated. GNSS data from the base is missing from 25 November to 16 December due to a power outage which led to the loss of the RTK GNSS mode and missing real-time baseline data after an automatic reboot. The RTK GNSS mode was manually re-established on December, 16, providing real-time data again. The near real-time GNSS SWE observations show a high level of agreement to the manual observations over the whole season, with differences below 50 mm w.e, an RMSE of 21 mm w.e, an MRB below 5%, and a cross correlation coefficient of 0.99 (Figure 5b,d). Settling of the snow was present after new snow accumulation with constant SWE and increased density. Melt was present at decreasing snow depth and decreasing SWE reference observations. The SWE observed by RTK GNSS refractometry followed the general trend of the snow scale observations (Figure 5a,c). However, bigger differences were present at the dry snow (up to 100 mm w.e.) period and emphasized at the wet snow period (up to 200 mm w.e.). The SWE observed by the snow scale deviates significantly compared to the manual and RTK GNSS observations. This could be due to an increased spatial snowpack heterogeneity during the snowmelt and different measurement locations of the snow scale and GNSS sensors. Lower correlation (0.96) and higher RMSE (68 mm w.e.) and MRB (24%) are present when compared to the snow scale observations. Regression coefficients are summarized in Table 3.

Noticeable is the big difference (approximately 200 mm w.e.) to the snow scale at the end of March and beginning of April which were caused by the over-measurement of the snow scale. Furthermore, a big difference (approximately 50 mm w.e.) to the snow scale and manual observation as well as the increased standard deviation of the GNSS-derived SWE is observable at the very end of December 2021. Heavy rainfall was observed that day, possibly influencing the RTK GNSS performance by increasing the path delay significantly. The constant part of the increased path delay may have been taken over by the receiver clock parameter and mitigated during double difference processing. Additionally, higher multipath due to the strong reflectivity of rain on snow and grounds could cause the strong deviations.

### 4.3. (Near) Real-Time SWE Estimation in the Field

Results of the mobile, low-cost, and (near) real-time GNSS refractometry snow monitoring setup (Section 2.3) at Davos Weissfluhjoch are demonstrated in Table 4. Three measurements were taken per hole. The RTK GNSS SWE based on the biased baseline Up component was measured down to a 170 cm depth and limited by the length of the ice drill. The estimated SWE is directly compared to the reference values of a close-by manual snow profile at Davos Weissfluhjoch. The SWE reference values were taken from the upper 170 cm of the manual snow profile. The SWE estimates from ambiguity fixed RTK GNSS solutions derived directly in the field show a high level of agreement with the reference measurements. Differences of 21 mm w.e. are close to the limit of RTK GNSS height solution accuracies (2–6 cm). It is noticeable that the ΔSWE is significantly increased in the case of float ambiguity solutions. It is thus important to fix the ambiguities before lowering the rover GNSS antenna in the hole to place the receiver below the snow surface for measuring the SWE.

## 5. Discussion

The present study investigated the applicability of the open-source GNSS post-processing software RTKLIB as well as the (near) real-time feasibility of the GNSS refractometry method using low-cost GNSS equipment. As the SWE induced GNSS path delay is highly collinear with the receiver clock and the height (Up) coordinate, the biased baseline Up component was successfully used for SWE estimation.

The seasonal SWE could be accurately determined over 15 min observation periods in post-processing using the GNSS refractometry method based on the biased GNSS baseline up component. In contrast to previous studies [23,24], the open-source GNSS post-processing software RTKLIB was applied to determine the SWE from a fixed, high-end GNSS refractometry snow monitoring setup with a very high temporal resolution (15 min). The software is fast, automatable, free and easy to use, and permits adequate setting options.

Moreover, the SWE was successfully estimated in (near) real time directly in the field with the developed mobile, low-cost GNSS snow monitoring setup. Fixed GNSS solutions are crucial so that the SWE can be estimated with a mean relative bias of 2.5% compared to manual observations. This is contradictory to a previous study which concluded that “resolving the GPS float ambiguities is shown to have no significant impact on the SWE estimation” [23]. There, the GNSS ambiguities were resolved over longer (daily) processing intervals, leading to an increased stability and accuracy of ambiguity float solutions. Accurate SWE time series can thus be monitored in near real time. However, there are limitations such as GNSS antenna phase center variations not matching with the changed incidence angles due to the refraction in snow. Real-time applications in shallow snowpacks could be limited due to the absolute positioning accuracy of RTK GNSS (2–3 cm). Relative positioning with subsequent filtering is, however, more accurate and could most likely allow an accurate estimation of changes in the SWE over time. Alternatively, the GNSS positioning accuracy is significantly increased (2–10 mm) in case of post-processing and small GNSS baselines, making accurate SWE estimations also possible in shallow snowpacks. Rainfall events are assumed to cause inaccuracies on the daily scale. Such deviations prevent or limit the use of daily GNSS SWE changes as a measure for mass gain or loss. It is noticeable that the noise of the unfiltered GNSS-derived SWE estimates is significantly reduced (approximately by a factor of 10) in case of post-processing compared to the near real-time application. Subsequent filtering of the near real-time SWE estimates is thus crucial to understand if daily GNSS-derived SWE variations are caused by accumulation/ablation or by the uncertainty of the SWE estimates from GNSS refractometry.

The present results show the possibility of SWE monitoring based on 1 Hz RTK GNSS refractometry Up component estimation. In this case, subsequent outlier screening and data filtering is significant for achieving accurate and reliable SWE time series. A highly resolved (10 min) SWE monitoring is very advantageous for new casting applications such as the temporal detection of precipitation or run-off events [33].

The SWE estimated with GNSS refractometry is currently only representative for a small spatial scale due to the point-wise measurement principle, estimating the SWE over an area of several square meters (up to 41 $m^{2}$) above the buried GNSS antenna. The low cost and maintenance, as well as the automatic processing of GNSS refractometry observations and the developed mobile setup allows distributed GNSS refractometry observations to increase the spatial resolution from point-wise to intermediate scales. The presented method is promising due to the small equipment size, cost, and installation effort. Low-cost GNSS systems can be used to monitor the SWE by estimating the biased GNSS baseline Up component between the base and rover antenna in RTK GNSS mode. The near real-time SWE retrieval method is automated, continuous, and nondestructive when installed during the snow-free period. The GNSS snow monitoring system, however, needs to be set up following best possible GNSS practices. Appendix A summarizes the main points which are important to enable successful GNSS observations.

Up to now, experimental GNSS refractometry snow monitoring systems consist of two GNSS antennas with one antenna buried underneath the snowpack and a close-by antenna which serves as a local reference above the snowpack. Among others, this very short GNSS baseline allows the mitigation of atmospheric GNSS path delays, such as the ionospheric and tropospheric delays. Tropospheric delays increase with higher elevation difference to the reference station and longer baselines. Ionospheric delays increase with longer baselines. They can be significantly reduced by using a linear combination of multi-frequencies. A national permanent GNSS network or virtual reference stations (VRS) could be utilized instead of the local GNSS reference for a distributed SWE monitoring in whole mountain catchment areas. Synergies can be used, and only the buried GNSS station needs to be installed in the snow monitoring site, reducing material, costs, and maintenance and allowing the installation in areas prone to avalanche danger. The feasibility of using long GNSS baselines will be investigated in a future study. The application of RTK GNSS refractometry in shallow snowpacks should be analyzed in more detail due to the limitation in the RTK GNSS positioning accuracy.

## Figures and Tables

**Table 1:** Regression coefficients for the comparison of the GNSS-derived SWE with each reference sensor’s data for 2016/17; b and m are the offset and slope of the linear regression fit, and n is the number of samples.

Reference Sensor	b (mm w.e.)	m	r	n	RMSE (mm w.e.)	MRB (%)
Manual	10	0.99	0.97	24	45.8	2.5
Snow scale	9	0.95	0.99	5627	34.0	10.6

**Table 2:** Applicability of RTKLIB for SWE estimation.

Pros	Cons
Open-source and free	Debugging
Fast data processing	No possibility to select GNSS solution type
User-friendly (GUI and command line versions)	Broadcast GNSS ephemerides needed
Automation possible	Noisy solutions
Import and export of processing options	
Multi-GNSS capability	

**Table 3:** Regression coefficients for the comparison of the GNSS-derived SWE with the manual (weekly) and snow scale (10 min) observations for 2021/22; b and m are the offset and slope of the linear regression fit, and n is the number of samples.

Reference Sensor	b (mm w.e.)	m	r	n	RMSE (mm w.e.)	MRB (%)
Manual	−8	1.04	0.99	21	21.4	4.5
Snow scale	−16	0.87	0.96	19,903	68.2	23.7

**Table 4:** Comparison of (near) real-time GNSS-derived SWE from the mobile, low-cost setup with the reference SWE at Davos Weissfluhjoch. Obs: observation.

Hole	Obs	Depth (cm)	SWE${}_{\mathrm{reference}}$ (mm w.e.)	SWE${}_{\mathrm{GNSS}}$ (mm w.e.)	Obs Interval (min)	RTK Solution	Δ SWE (mm w.e.)
1	1	170	571	550	15	fixed	21
	2	170	571	550	15	fixed	21
	3	170	571	550	15	fixed	21
2	1	170	571	850	13	float	279
	2	170	571	850	15	float	279
	3	170	571	550	20	fixed	21

## Data Availability

Collected and analyzed research data are made publicly available at Zenodo [28,29]. Python scripts for processing, analyzing, and visualizing the data is available on Github [31,34].

## Appendix A. Best Practice and Limitations

GNSS antennas which are buried in a snowpack are exposed to an environment they are not designed for. Observed signals are reflected at the air/snow surface and attenuated within the snowpack. This leads to a changed GNSS observation geometry and reduced signal strengths, especially in a wet snow environment. To minimize the SWE or coordinate estimation errors, it is important to reduce other distracting factors by ensuring an optimal monitoring setup:

- Good satellite visibility and observation geometry; satellites from low to high elevations from different azimuth angles should be trackable. Narrow mountain valleys or forests strongly decrease the sky view and therefore deteriorate the observation geometry.
- Keep mandatory obstacles in the North of the antennas (Northern Hemisphere) or in the South (Southern Hemisphere) as less GNSS observations are influenced due to the orbit inclination and the satellite distribution.
- Prevent or minimize metallic structures around the GNSS setup as they cause reflections and delays of the electromagnetic GNSS signals.
- Place the GNSS antenna near field outside metallic structures (e.g., mounting pole).
- Use similar GNSS equipment for the base and rover station; similar GNSS antennas and receiver.
- The base and rover antennas should be oriented equally to minimize effects of the antenna gain patterns; alternatively, use antenna phase center offset and variation files and orient the GNSS antennas to North. Typically, these antenna calibration files are only available for high-end GNSS antennas.
- Protect the setup from wildlife and human interactions.
- Take measures to limit snow accumulation on the top antenna.
- Use short baselines between the base and rover to mitigate atmospheric effects due to the correlation between the troposphere and the station height parameters.
- Keep the baseline physically fixed due to the collinearity of the station height and the SWE parameters.
- Use a base plane for the antennas to reduce multipath reflections from below.

## Appendix B. Processing Configuration

### Appendix B.1. RTKLIB Post Processing Command

The GNSS refractometry post-processing is automated using the RTKLIB command line tool rnx2rtkp:

rnx2rtkp -k config-day.txt -ti interval -o out.pos rover.yyO base.yyO day.nav day.sp3

specifying the options and input files:
-k: configuration file (config-day.txt)
-ti: time interval (1 s)
-o: output file (out.pos)
base.yyO: RINEX file of base receiver
rover.yyO: RINEX file of rover receiver
day.nav: RINEX broadcast navigation file
day.sp3: precise ephemerides of GNSS satellites

### Appendix B.2. Emlid Reach M2 RTK Configuration

The Emlid Reach M2 receivers are configured in RTK GNSS positioning mode for the RTK GNSS refractometry SWE estimation directly in the field (Section 3.2.3). The base station data (server) is transmitted to the rover (client) via TCP/IP and RTCM3 messages. The observation configuration is listed in Table A1.

**Table A1:** RTK GNSS refractometry observation settings.

Property	Base	Rover
Positioning mode	static	static
Elevation mask	0	0
SNR mask	35	25
GNSS systems	GPS, GLONASS, GALILEO, BEIDOU	GPS, GLONASS, GALILEO, BEIDOU
Update rate	1 Hz	1 Hz
Solution output	-	ENU

### Appendix B.3. RTKLIB and Emlid Reach Solution File Format

The GNSS solution file (.POS) generated by RTKLIB post-processing equals the format of the RTK logged baseline output file (.ENU) from Emlid Reach RTK receivers. Unfortunately, no header is present in the .ENU files, and the column names are given in Table A2. Sample .ENU files and the SWE visualization code is provided by [34].

**Table A2:** Column names in the output .ENU solution files, generated by the buried Emlid Reach M2 receiver. The baseline components in East, North, Up (e,n,u) are given in WGS84. The quality flag (q) indicates the GNSS solution type: 1:fix; 2:float; 3:sbas; 4:dgps; 5:single; 6:ppp; ns indicates the number of satellites used for the GNSS solution.

UTC	e	n	u	q	ns	sde	sdn	sdu	sden	sdnu	sdue	age	ratio
(date time)	(m)	(m)	(m)			(m)	(m)	(m)	(m)	(m)	(m)	(s)	
